# Bio-Herbicidal Potential of Nanoemulsions with Peppermint Oil on Barnyard Grass and Maize

**DOI:** 10.3390/molecules27113480

**Published:** 2022-05-28

**Authors:** Magdalena Rys, Małgorzata Miastkowska, Elżbieta Sikora, Anna Łętocha, Agnieszka Krajewska, Agnieszka Synowiec

**Affiliations:** 1 The Franciszek Górski Institute of Plant Physiology, Polish Academy of Sciences, Niezapominajek 21, 30-239 Krakow, Poland; m.rys@ifr-pan.edu.pl; 2 Faculty of Chemical Engineering and Technology, Institute of Organic Chemistry and Technology, Cracow University of Technology, 31-155 Krakow, Poland; malgorzata.miastkowska@pk.edu.pl (M.M.); elzbieta.sikora@pk.edu.pl (E.S.); anna.letocha@doktorant.pk.edu.pl (A.Ł.); 3 Department of Biotechnology and Food Science, Lodz University of Technology, 90-530 Lodz, Poland; agnieszka.krajewska@p.lodz.pl; 4 Department of Agroecology and Crop Production, The University of Agriculture in Krakow, 31-120 Krakow, Poland

**Keywords:** Eco-Polysorbate 80, phytotoxicity, isothermal calorimetry, polydispersity index, FT-Raman spectroscopy, relative water content, Chlorophyll *a* fluorescence

## Abstract

Bio-based nanoemulsions are part of green pest management for sustainable agriculture. This study assessed the physicochemical properties and the herbicidal activities of the peppermint essential oil nanoemulsions (PNs) in concentrations 1.0–10% stabilized by Eco-Polysorbate 80 on germinating seeds and young plants of maize and barnyard grass. Based on the design of experiment (DOE) results, the final nanoemulsion formulations were obtained with 1, 1.5, 2, and 5% of essential oil concentration. Biological analyses were conducted to select the most promising sample for selective control of barnyard grass in maize. Seedlings growing in the presence of PNs displayed an overall inhibition of metabolism, as expressed by the calorimetric analyses, which could result from significant differences in both content and composition of carbohydrates. Concentration–response sub estimation showed that leaf-sprayed concentration of PN causing 10% of maize damage is equal to 2.2%, whereas doses causing 50% and 90% of barnyard grass damage are 1.1% and 1.7%, respectively. Plants sprayed with PN at 5% or 10% concentration caused significant drops in relative water content in leaves and Chlorophyll *a* fluorescence 72 h after spraying. In summary, peppermint nanoemulsion with Eco-Polysorbate 80 at 2% concentration is a perspective preparation for selective control of barnyard grass in maize. It should be analyzed further in controlled and field conditions.

## 1. Introduction

Agriculture and the food industry are part of sustainable development and human health challenges. A significant portion of sustainable agriculture is the development of natural, environmentally friendly methods of managing agricultural pests, which would successfully replace synthetic pesticides. For that reason, nanotechnology in agriculture has a significant role. Agricultural nanotechnology applications include reducing the number of distributed pesticides, minimizing nutrient losses in fertilization, increasing crop yield, and producing nano-pesticides that can effectively protect crops. In addition, nanomaterials as unique carriers of agrochemicals facilitate site-directed, controlled delivery of nutrients with increased crop protection and productivity without soil and water decontamination and protection against several insect pests and microbial diseases [1,2]. Nanoformulations facilitate using the minimum effective concentration of the active ingredients in the formulation at the target sites and the active ingredients’ controlled release in the root zones or inside the plants without compromising effectiveness. Conventional pesticide or herbicide formulations reduce the water solubility of pesticides and damage other organisms, leading to increased pesticide resistance in target organisms. Nanoformulations also overcome these limitations [2].

Essential oils (EOs), natural compounds of plant origin, could serve as an important source of active compounds for replacing synthetic pesticides with the help of nanotechnological methods. Previous research has proved EOs as compounds for the production of botanical pesticides, especially botanical insecticides [3] and botanical herbicides [4], which are of interest to agriculture since they show remarkable results in inhibiting the growth of weeds and additionally are biocompatible and environmentally friendly; these are their advantages over synthetic herbicides. It was proved that essential oils containing monoterpene alcohols or oxygenated monoterpenes as the main compounds produce effective botanical herbicides, mainly against annual dicotyledonous weeds in their early growth stages [4,5,6]. One of the prospective oils for these uses is a peppermint oil (Menthae piperitae aetheroleum), distilled from the aerial parts of *Mentha × piperita* (L.) herb, botanical family Labiatae. The content of EOs in the peppermint herb originating from Poland equals 2.1–2.5% [7]. The oil is colorless, pale yellow, or greenish-yellow, with a characteristic odor and taste followed by a cold sensation. The main compounds of peppermint oil are menthol and menthone, which constitute 14–32% and 30–55%, respectively, of all oil compounds. The other key compounds of the oil include cineol, isomenthone, menthyl acetate, limonene, pulegone, and carvone [8]. The peppermint oil applied in numerous in vitro research has displayed pesticide activities, showing that this EO is useful for further in situ experiments [9]. Leaf-applied EO displays a contact action, and damages caused by the EO are visible as early as a few hours following its application [10,11].

However, there are some barriers to using essential oils as botanical herbicides as they decompose easily due to direct exposure to heat, light, or oxygen [12,13]. Therefore, to effectively use the herbicidal potential of EO, formulations that are stable under changing environmental conditions are still needed. One of the alternative forms of application of essential oils is nanoformulation, including nanoemulsion, which effectively increases active substances’ physical stability, reduces volatility, and prevents interactions with the environment. The use of nanoemulsions for agricultural purposes is further advantageous in that they mix easily with soil particles and inhibit the growth of weeds resistant to commercial herbicides [14,15,16].

Peppermint oil-based nanoemulsions are known from the literature [17,18,19,20]. In previous studies, the oil-in-water emulsion of peppermint oil was applied either with the fatty acid methyl esters of natural origin [21] or with a commercial multifunctional adjuvant for synthetic herbicides containing 80% of fatty acid methyl esters of oilseed rape oil [22]. All the emulsions were sprayed on the leaves of barnyard grass (*Echinochloa crus-galli*) and maize (*Zea mays*). As a result, the authors observed significant damages caused by the emulsions. However, the selectivity effect was not achieved, as both maize and barnyard grass were severely injured after spraying, with leaf surface damages of 30–40% for both tested plants [21,22]. Moreover, significant inhibition of photosynthetic efficiency of leaves and perturbations of metabolic activity of both barnyard grass and maize followed the emulsion spraying [22].

To the best of our knowledge, there is no report on the preparation of peppermint EO nanoemulsion stabilized by Eco-Polysorbate 80, which was formed by reacting 100% bio-based ethylene oxide with 100% bio-based sorbitan oleate [23,24]. It makes our formulation 100% eco-friendly, environmentally safe, and biodegradable. For that reason, this study aimed to assess the physicochemical properties and the herbicidal activities of the nanoemulsion of peppermint essential oil stabilized by Eco-Polysorbate 80 on germinating seeds and young plants of maize (*Zea mays* L.) and barnyard grass (*Echinochloa crus-galli* (L.) Beauv.).

## 2. Results

### 2.1. Chemical Composition of Peppermint Essential Oil Used in the Study

Peppermint essential oil composition was analyzed using GC-FID-MS analysis. Twenty-seven volatile constituents were identified, corresponding to 98.2% of total constituents in this oil (Table 1). The main group of compounds was oxygenated monoterpenes, mainly alcohols and aldehydes. Menthol (44.2%), menthone (27.4%), and their isomers such as iso menthone (4.6%) and neomenthol (2.5%) were present in the greatest amounts. The qualitative and quantitative composition complies with the European Pharmacopeia 7.0 [25].

### 2.2. The Influence of Input Parameters on Properties of Obtained Nanoemulsions

**Figure 1:**
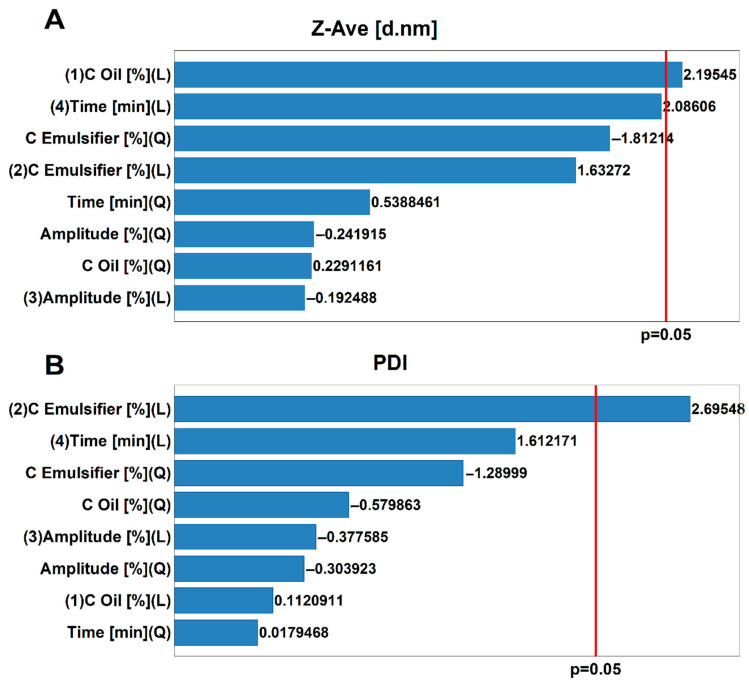
Pareto charts for the influence of input parameters (oil concentration, emulsifier concentration amplitude, sonification time) on (**A**) average droplet size of nanoemulsions (d. nm), (**B**) polydispersity Index (PDI).

Statistical analysis was performed based on a one-way analysis of variance (ANOVA). To evaluate the significance of the differences, an F-test was used. It was checked whether the input parameters significantly affect the output parameters of the peppermint nanoemulsions (PNs). The input parameters included oil concentration (COil (%)), the emulsifier concentration (CEmulsifier (%)), the amplitude, and the sonification time. The group of initial parameters included the particle size (nm), polydispersity index (PDI), viscosity (mPa · s), and stability. A value of *p* < 0.05 was established as significant in all cases. Given the profiles of the utility function to certain independent parameters, thanks to which it was possible to determine changes, >0.05 was established as significant in all the PNs. Figure 1 presents the Pareto charts for input parameters of PNs. Parameters that are statistically significant for the established *p*-value (*p* < 0.05) are marked with a red line. From the Pareto charts, it is concluded that neither the viscosity nor the stability of PNs depends on any of the input parameters mentioned. The parameter that has a statistically significant impact on the particle size of PNs is the oil concentration (Figure 1A). On the other hand, as a linear function, the concentration of the emulsifier is a parameter that has a statistically significant influence on the polydispersity index (PDI) (Figure 1B).

**Figure 2:**
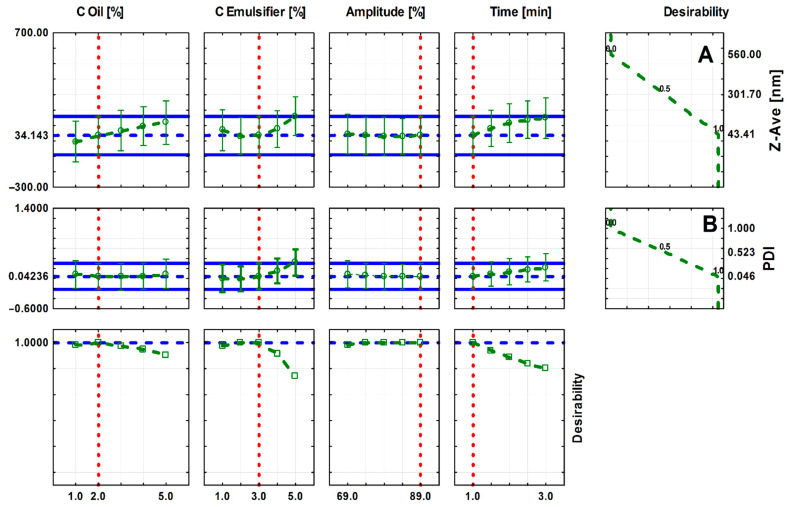
Approximation profiles for the influence of input parameters on (**A**) an average droplet size (nm) of nanoemulsions (nm), (**B**) polydispersity index (PDI).

The approximation profiles are presented in Figure 2. Based on an assessment of their approximation profiles, the influence of independent parameters on dependent variables and the determination of the specific values of input parameters ensure that reaching the desired values of the output parameters is possible. The smallest droplet size of PNs and the smallest PDI values are desirable. The PN characterized by the low PDI, and a monomodal particle size distribution is considered more kinetically stable. The PDI represents the distribution of particle size fractions within a given sample. The numerical value of PDI ranges from 0.0 (for a perfectly uniform sample concerning the particle size) to 1.0 (for a highly polydisperse sample with multiple particle size fractions).

The analysis of the approximation profiles (Figure 2) shows that peppermint nanoemulsions (PNs) with the smallest particle size (approx. 34 nm) and the smallest polydispersity index (PDI = 0.0423) are obtained for an oil concentration of 2%, intermediate emulsifier concentration of 3%, the minimum ultrasonication time = 1 min, and the maximum amplitude value (89%). The graphs also show that the average size of the PNs particles increases with the increase in the oil concentration and the ultrasonication time. In the case of an emulsifier concentration, the particle size decreases to the intermediate value of the emulsifier concentration (3%) and then begins to increase. Moreover, an increase in the PDI value is observed with an increase in the emulsifier concentration and the sonication time increase.

**Figure 3:**
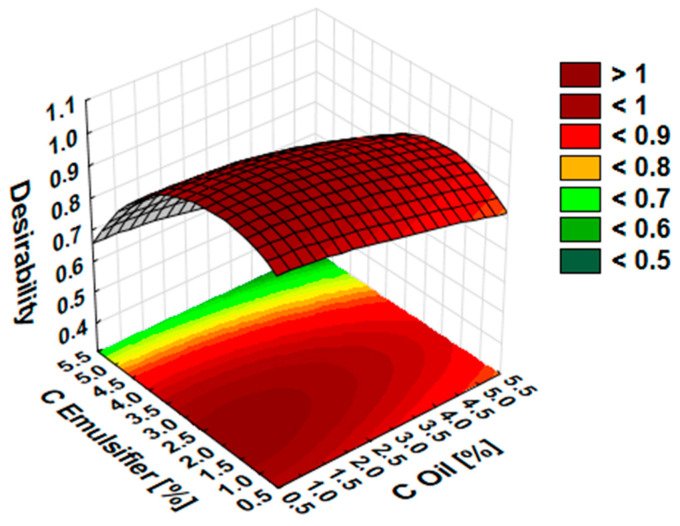
Saddle plots for desirability for essential oil and emulsifier concentration.

The data presented in Figure 3 suggested that to obtain nanoemulsion with desired physicochemical parameters, such as the smallest droplet size of internal phase and the smallest PDI value, emulsifier and essential oil concentration should be in the range from 1.0 to 3.5% and 1.0 to 5.0%, respectively.

Based on the design of experiments (DOE) results, samples of nanoemulsions containing peppermint oil were prepared (Table 2). Different ratios of the essential oil (O) to surfactant (S) were tested, and finally 1:1 (*v*/*v*) O:S ratio was chosen. All samples were prepared using the minimum ultrasonication time (t = 1 min) and the maximum amplitude value (89%). Stability analysis showed no significant changes in droplet size and polydispersity index during 30 days of storage and after stability (Table 2). Moreover, all the samples are characterized by a very small droplet size of around 100 nm and a very low PDI index (PDI < 0.1) which are desired values of the output parameters (Table 2).

**Figure 4:**
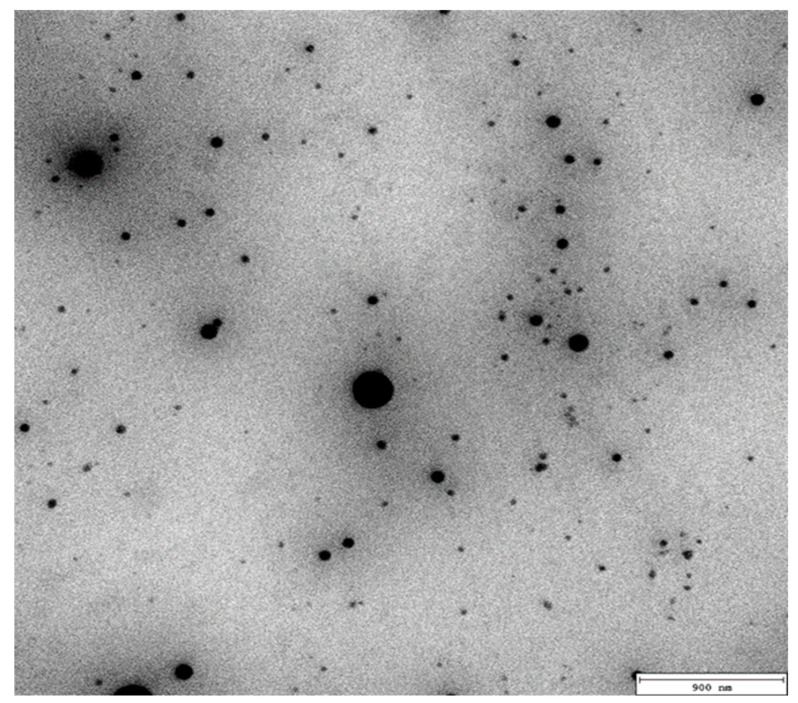
Transmission electron micrograph of peppermint oil nanoemulsion (5% *v*/*v*).

Morphology and size of nanoemulsions were also analyzed by transmission electron microscopy (TEM). The TEM image (Figure 4) of the nanoemulsion containing 5% of peppermint oil confirmed that droplets were spherical in the nano-size range.

### 2.3. Metabolic Activity of Maize and Barnyardgrass Seedlings Treated with PNs

Thermal power is emitted by plant tissue during germination and growth.

**Figure 5:**
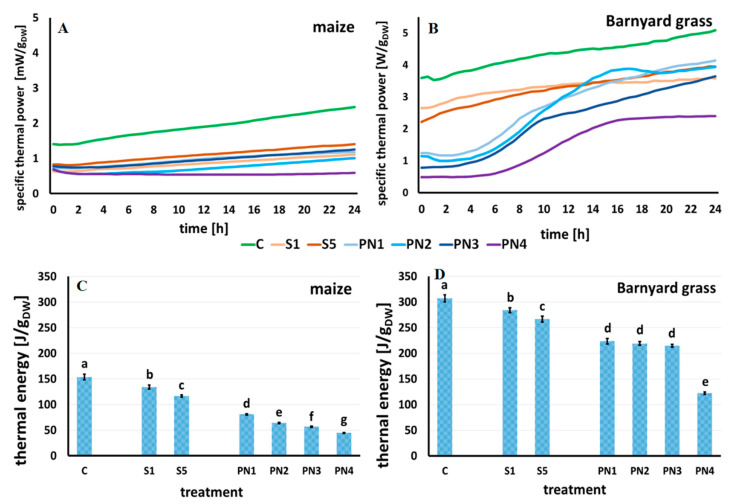
The specific thermal power curves of the maize (**A**) and barnyard grass (**B**) seedlings growing on the surfactants (S1 light orange line and S5 dark orange line), nanoemulsions with peppermint oil (PN1 light blue line, PN2 cyan line, PN3 dark blue line, PN4 violet line) and water (C green line). The total heat emitted by maize (**C**) and barnyard grass (**D**) seedlings growing on the surfactants S1 (1% *v*/*v*) and S5 (10% *v*/*v*), nanoemulsions with peppermint oil PN1 (1% *v*/*v*), PN2 (1.5% *v*/*v*), PN3 (2% *v*/*v*), PN4 (5% *v*/*v*), and water (C). Mean values ± SD. The letters a–g indicate statistically significant differences. Values indicated by the same letters did not differ significantly at *p* ≤ 0.05 according to Duncan’s test (within the tested species), *n* = 10.

In the water-treated (control) seedlings of maize (Figure 5A) and barnyard grass (Figure 5B), the obtained specific thermal power curves increased linearly during the measurement for 24 h, reaching the values of 2.45 mW·g_DW_^−1^ for maize and 5.09 mW·g_DW_^−1^ for barnyard grass.

The shape of thermal power curves for maize under different treatments showed a linear increase. For each treatment, the beginning value of specific thermal power was ~0.75 mW·g_DW_^−1^. After 24 h of measurements, the highest value of specific thermal power was observed for the seedlings growing on surfactant S5 (10% *v*/*v*)—1.41 mW·g_DW_^−1^, and slightly lower values were recorded for the seedlings growing on nanoemulsions PN1 (1% *v*/*v*) and PN3 (2% *v*/*v*)—1.18 mW·g_DW_^−1^ and 1.25 mW·g_DW_^−1^, respectively. The lowest specific thermal power value was noted for the seedlings, which were grown on PN4 (5% *v*/*v*)—0.68 mW·g_DW_^−1^, a value lower than the beginning value, at the start of measurement (Figure 5A).

In the case of thermal power curves for the seedlings of barnyard grass, which were grown on surfactants S1 and S5, they were similar, and the specific thermal power values obtained at the end of measurements were 3.57 mW·g_DW_^−1^ for S1 and 3.94 mW·g_DW_^−1^ for S5. For the seedlings growing on nanoemulsions PN1 (1% *v*/*v*) and PN3 (2% *v*/*v*), specific thermal power values were observed in the measuring time, with a slight inhibition after approximately nine and ten hours for PN1 and PN3, respectively. The final specific thermal power values were 4.14 mW·g_DW_^−1^ and 3.65 mW·g_DW_^−1^ for PN1 and PN3, respectively. In the case of barnyard grass growth on PN2 (1.5% *v*/*v*) and PN4 (5% *v*/*v*), the maximum peak of specific thermal power was observed after sixteen hours of continuous growth. After that time, a decrease in the specific thermal power values was noted, of 3.94 mW·g_DW_^−1^ and 2.40 mW·g_DW_^−1^ at the end of measurements for PN2 and PN4, respectively (Figure 5B).

The effect of surfactants (S) and peppermint nanoemulsions (PNs) at various concentrations on the overall metabolism of the plant seedlings can be illustrated by the amount of total thermal energy that was emitted during their growth at the time of measuring, which is also known as the seedling growth enthalpy. The value of thermal energy is equal to the area under the specific thermal power curve and was calculated by integrating these curves.

In maize and barnyard grass, the thermal energy was the highest for the control seedlings, ~154 J·g_DW_^−1^ and ~307 J·g_DW_^−1^, respectively (Figure 5C,D). Surfactants in both concentrations (S1 and S5) significantly decreased the thermal energy values in the tested species compared to the control. For maize, this was a 13% reduction for the S1 and 24% for the S5 (Figure 5C). In barnyard grass, there was a reduction of 7.5% for the S1 and 13% for the S5 (Figure 5D).

In maize, a significant decrease in thermal energy was observed for the seedlings growing on PNs. The higher the oil concentration in the PN, the lower the value of thermal energy, with the control seedlings and those grown on both surfactants. Compared to the control, there was a 47%, 58%, 63%, and 71% decrease for the maize seedlings growing on PN1, PN2, PN3, and PN4, respectively (Figure 5C). For barnyard grass, the amount of thermal energy emitted during the growth on the PN1, PN2, and PN3 did not differ statistically from each other. The lowest value of thermal energy was recorded for the seedlings growing on PN4 (~122 J·g_DW_^−1^), which was significantly different from all the other treatments (Figure 5D).

### 2.4. FT-Raman Spectroscopy of Maize and Barnyardgrass Seedlings Treated with PNs

**Figure 6:**
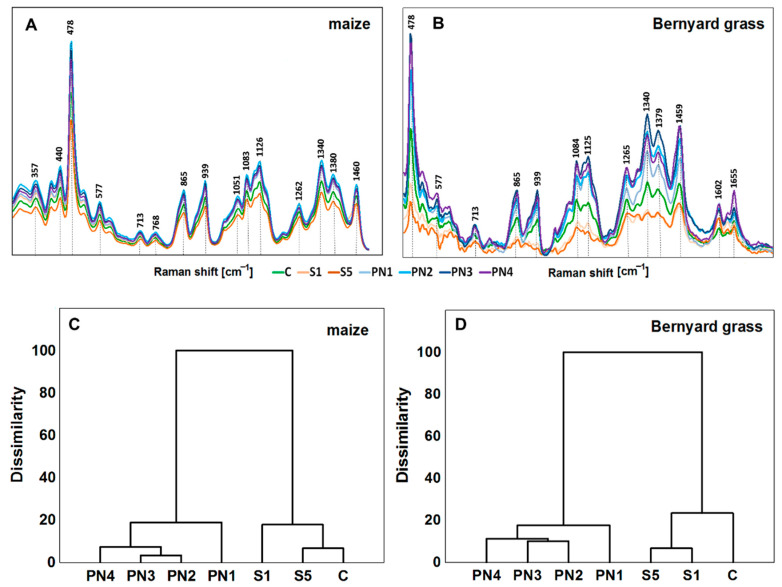
FT-Raman spectra show the chemical composition of seedling’s endosperm of maize (**A**) and barnyard grass (**B**) that were growing on the surfactants (S1, light orange line, and S5, dark orange line), nanoemulsions with peppermint oil (PN1, light blue line; PN2, cyan line; PN3, dark blue line; PN4, violet line) and water (C, green line). The mean values were based on six repetitions. Hierarchical cluster analysis of the FT-Raman spectra of the maize (**C**) and barnyard grass (**D**). Surfactants S1 (1% *v*/*v*) and S5 (10% *v*/*v*), nanoemulsions with peppermint oil PN1 (1% *v*/*v*), PN2 (1.5% *v*/*v*), PN3 (2% *v*/*v*), PN4 (5% *v*/*v*), and water (C—control).

The spectral region between 350 and 1600 cm^−1^ was selected for the analysis as the most interesting and represents the vibration modes of various carbohydrate bonds that refer to the storage nutrients in the endosperm of maize and barnyard grass (Figure 6A,B, respectively). The summaries of most characteristic bands are also described in Table 3. 

The bands on Raman spectra can be assigned to carbohydrates (410–1265 cm^−1^), aliphatic hydrocarbons (CH_2_ and CH_2_/CH_3_ vibrations), carbohydrates (1340–1460 cm^−1^), and lignins (1601–1665 cm^−1^). The most intense bands, observed at 478 cm^−1^ and the band at 940 cm^−1^, are typical for starch. The remaining bands, visible in the spectrum, are related to carbohydrates in plant tissues, particularly amylase and amylopectins (Table 3).

A hierarchical cluster (a similarity) analysis systematically grouped the studied objects into clusters to find the meaningful and systematic differences in the measured FT-Raman spectra. The cluster analysis was performed in the band range from 280 to 1500 cm^−1^ for the endosperm of maize seedlings and in the band range from 450 to 1800 cm^−1^ for the endosperm of barnyard grass seedlings (Figure 6C,D, respectively). Statistically, distinct discrimination was achieved for both tested species for two groups. The first group consisted of the control seedlings and those which grew on surfactants (S1 and S5). In contrast, the second group included the seedlings exposed to nanoemulsions with peppermint oil in all the tested concentrations (PN1, PN2, PN3, and PN4). It was shown that the chemical composition of endosperms of maize and barnyard grass seedlings differed significantly in both content and composition of carbohydrates. These differences depended on the PNs concentration. 

### 2.5. Herbicidal Effects of Leaf-Sprayed PNs on Maize and Barnyard Grass

**Figure 7:**
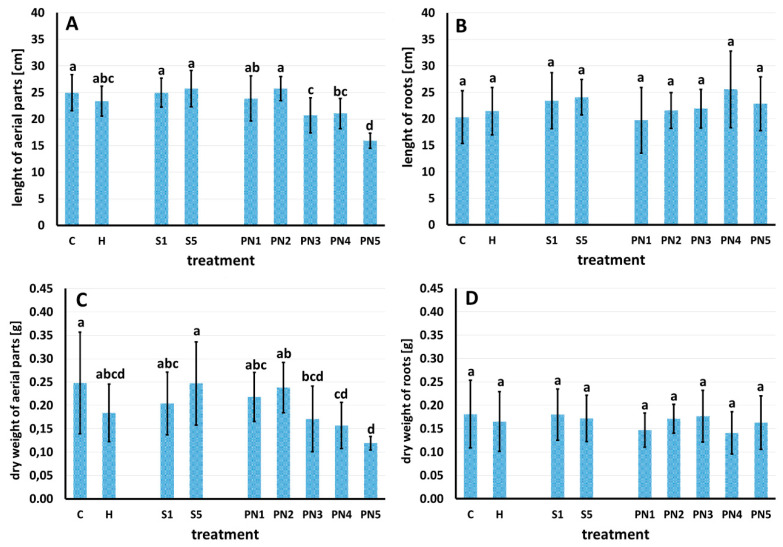
Length of aerial parts (**A**), length of roots (**B**), dry weight of aerial parts (**C**), and dry weight of roots (**D**) of maize seven days after spraying with water—C, herbicide—H, surfactants—S1 (1% *v*/*v*) and S5 (10% *v*/*v*), and nanoemulsions with peppermint oil PN1 (1% *v*/*v*), PN2 (1.5% *v*/*v*), PN3 (2% *v*/*v*), PN4 (5% *v*/*v*), and PN5 (10% *v*/*v*). Bars represent mean value ± SD. The letters a–d indicate statistically significant differences. Values indicated by the same letters did not differ significantly at *p* ≤ 0.05 according to Duncan’s test (within the tested species), *n* = 4.

Seven days after spraying, significant changes in the length and dry weight of the aerial parts of maize (Figure 7A,B), and no effect on the length and dry weight of maize roots, were observed (Figure 7C,D).

The first group of maize plants, with the longest aerial parts, included control sprayed with water (C), surfactants S1 and S5 (1% and 10% *v*/*v*), and nanoemulsion PN2 (1.5% *v*/*v*). The second group, but non-significantly different from the first one, consisted of maize sprayed with herbicide (H) and nanoemulsion PN1 (1% *v*/*v*). Of a significantly reduced length of the aerial parts, the third group was maize sprayed with nanoemulsions PN3 and PN4 (2% and 5% *v*/*v*); the shortest aerial parts of maize were recorded after spraying it with the nanoemulsion PN5 (10% *v*/*v*). The aerial parts of the maize sprayed with PN5 were 36% shorter than those of the control C (Figure 7A). The dry weight of the aerial parts of maize was generally in line with their length. The maize plants sprayed with water (C) and surfactant S5 displayed the highest dry weight of the aerial parts. The lowest weight of aerial parts was recorded for maize sprayed with PN4 and PN5; it was lower by 36–48% than the C and S5 maize. At the same time, the dry weight of maize sprayed with PN5 was similar to the dry weight of maize sprayed with herbicide (H) and PN4 and PN3 (Figure 7B).

**Figure 8:**
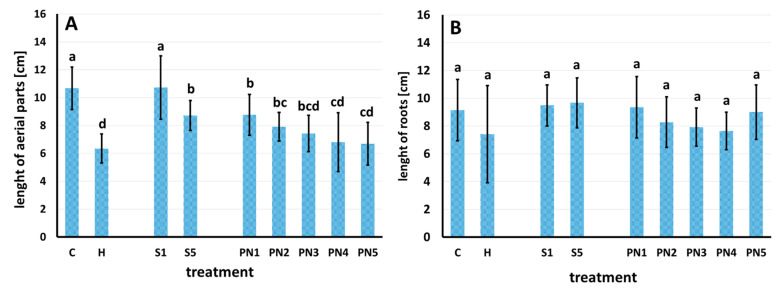
Length of aerial parts (**A**) and length of roots (**B**) of barnyard grass seven days after spraying with water—C, herbicide—H, surfactants—S1 (1% *v*/*v*) and S5 (10% *v*/*v*), and nanoemulsions with peppermint oil PN1 (1%*v*/*v*), PN2 (1.5% *v*/*v*), PN3 (2% *v*/*v*), PN4 (5% *v*/*v*), and PN5 (10% *v*/*v*). Bars represent mean value ± SD. The letters a–d indicate statistically significant differences. Values indicated by the same letters did not differ significantly at *p* ≤ 0.05 according to Duncan’s test (within the tested species), *n* = 4.

Spraying with the PNs caused significant changes in the length of barnyard grass aerial parts (Figure 8A) but not its roots (Figure 8B). Barnyard grass sprayed with water (C) and S1 had the longest aerial parts; these plants differed significantly from all the other treatments. The barnyardgrass sprayed with S5, PN1, and PN2 was the second homogeneous group with the aerial parts significantly longer than the PN4, PN5, and H, constituting the third group with the shortest aerial parts length, 36–40% shorter than the C plants. Barnyard grass sprayed with the PN3 did not differ significantly from the second and third groups, and compared to C, it had the aerial length shorter by 30% (Figure 8A). 

A dose–response analysis carried out for the aerial parts damage (necrosis) on a 0–100% scale allowed us to estimate the effective doses (ED) of PNs, causing 10 and 50% of maize necrosis (ED10 and ED50) and 50 and 90% of barnyard grass necrosis (ED50 and ED90) (Table 4). In the case of maize, ED10 was equal to 2.2%, and ED50 was five times higher and equal to 10%. On the other hand, in the case of barnyard grass, the estimated ED50 was 1.1%; and the estimated ED90 was 1.7%. The representative photos of damages caused to plants treated with the PNs are presented in Supplementary Figure S1.

**Figure 9:**
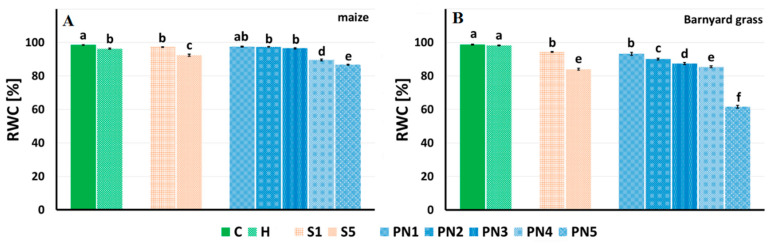
Relative water content (RWC) of maize (**A**) and barnyard grass (**B**) that were growing on the surfactants S1 (1% *v*/*v*) and S5 (10% *v*/*v*), nanoemulsions with peppermint oil PN1 (1% *v*/*v*), PN2 (1.5% *v*/*v*), PN3 (2% *v*/*v*), PN4 (5% *v*/*v*), and PN5 (10% *v*/*v*), herbicide (H) and water (C) calculated as (fresh mass—dry mass)/(turgid mass—dry mass)] × 100. The letters a–f indicate statistically significant differences. Values indicated by the same letters did not differ significantly at *p* ≤ 0.05 according to Duncan’s test (within the tested species), *n* = 10.

The relative water content (RWC) in maize leaves changed 72 h after spraying (Figure 9A). A homogenous RWC group was noted for H, S1, PN2, and PN3 treatments, significantly lower than C. The lowest RWCs were noted for the treatments S5, PN4, and PN5. Compared to the C, the PN4 and PN5 treatments decreased the RWC of maize by 9% and 12%, respectively (Figure 9A).

The RWC values for barnyard grass were the highest for C and H and amounted to over 98% (Figure 9B). The S1 and S5 treatments decreased the RWC by 5% and 18%, respectively, compared to the C. All the PNs significantly decreased the RWC values compared to the C. The higher the concentration of the peppermint oil in PN (1–10%), the statistically lower the RWC value (93–61%). The PN5 lowered the RWC by over 38% compared to the C.

**Figure 10:**
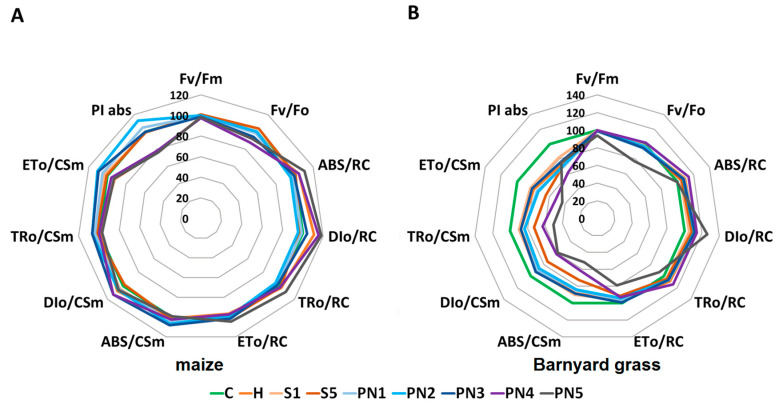
Values of selected Chl *a* fluorescence parameters of maize (**A**) and barnyard grass (**B**). C—water control, H—herbicide, surfactants S1 (1% *v*/*v*) and S5 (10% *v*/*v*), nanoemulsions with peppermint oil PN1 (1% *v*/*v*), PN2 (1.5% *v*/*v*), PN3 (2% *v*/*v*), PN4 (5% *v*/*v*), and PN5 (10% *v*/*v*).

The efficiency of photosystem II (PSII) was described based on the fast-kinetic Chlorophyll *a* fluorescence and shown on the spider graphs, where the values for the control plants (C) are presented as 100% (Figure 10A,B) and in Supplementary Material—Tables S1 and S2.

For maize, the values of the majority of fluorescence parameters were not affected following spraying with S or PNs. The maximum quantum yield of the photosystem II (Fv/Fm) and the maximum quantum yield of primary photochemistry (Fv/F0) were similar for almost all the treatments, except PN4, which differed significantly from C and was reduced by more than 2% and 12%, respectively (Figure 10A, Table S1). A similar situation was observed for the parameters that describe the specific energy fluxes calculated per reactive center (RC), such as the energy absorbed by the antennas (ABS/RC), the energy dissipated as heat (DIo/RC), the energy transferred to the RC (TRo/RC), and the energy flow to the electron-transport chain (ETo/RC), where the significant increases were observed for the PN5 treatments only, in comparison to C and H. The increases were over 10% for ABS/RC, 18% for DIo/RC, almost 9% for TRo/RC, and over 4% for ETo/RC. The parameters that are connected with the phenomenological fluxes per cross-section (CS) such as the energy absorption by the antenna system (ABS/CSm), the energy trapped in the reaction center (TRo/CSm), the energy flux to the electron-transport chain (ETo/CSm), and the energy that dissipated as heat (DIo/CSm), were similar for S and PNs treatments, compared to C and H. The performance index (PI) was strongly reduced only for the PN4 (by 22%) and PN5 (by 24%) treatments compared to C, and both these values were similar to H.

For barnyard grass, the Fv/Fm and Fv/F0 parameters reduced significantly, compared to C and H only at the PN5 treatment—by 6% and 22%, respectively (Figure 10B, Table S2). Among the parameters that describe the specific energy fluxes calculated per reactive center (RC), significant increases of ABS/RC and TRo/RC were observed for the PN4 treatment only, by 14%, compared to C. A significant increase by 26% for DIo/RC and a significant decrease of 21% for ETo/RC, was observed only for the PN5 treatment, compared to C. More significant changes were noted in the phenomenological energy fluxes per excited cross-section (CSm). Compared to C, significant drops in all these parameters were observed for the S5, PN4, and PN5 treatments. Moreover, in the case of the ETo/CSm parameter, its values significantly dropped for all H, S, and all PNs treatments, compared to C. The performance index (PI) of barnyard grass was also significantly reduced, compared to C, by all the treatments, but a major decrease was noted for the PN4 treatment, by 38%.

## 3. Discussion

The application of nanoscale delivery systems may increase passive cellular absorption mechanisms, thus reducing mass transfer resistance and increasing the biological activity of essential oil [32,33]. Considering the above, when planning this experiment, it was assumed that the smallest droplet size of nanoemulsions and the smallest polydispersity index (PDI) value is the most desirable parameters to increase the biological activity of essential oil. The smaller droplets of nanoemulsion, the larger surface area, which may influence the essential oil (active substance) transport to plants’ cells and eventually lead to cell death [34,35]. 

Based on the design of experiments (DOE) results, input parameters that significantly impact the particle size and the polydispersity index of peppermint oil nanoemulsions (PNs) are found in the essential oil and emulsifier concentration, respectively. The data presented at approximation profiles and saddle plots suggested that to obtain nanoemulsion with desired physicochemical parameters, such as the smallest droplet size of internal phase and the smallest PDI value, emulsifier and essential oil concentration should be in the range from 1.0 to 3.5% and 1.0 to 5.0%, respectively. The final nanoemulsion formulations were obtained, considering all this information. Moreover, we observed that adding peppermint oil with up to 5% concentration to the formulations probably reduced the formulation’s droplet size. This may be since some essential oil components have a co-surfactant-like structure and may decrease the interfacial tension of an emulsion system [36]. Those data agree with the research carried out by Rehman et al. [37]. They observed that peppermint oil-loaded nanoemulsions showed a significant reduction in mean droplet size and polydispersity index up to a critical loading concentration of essential oil which was 5.5%. An increase in droplet size was observed above that concentration, and finally, sample destabilization. The biological analysis has been conducted in the next stage of studies to select the most promising sample among final formulations for selective control of barnyard grass in maize.

The life processes of the seedlings of maize and a monocotyledonous weed—barnyard grass—were monitored using isothermal calorimetry to track changes in the amount of energy they release when treated with the surfactants and the peppermint nanoemulsions. The calorimetric method is used to determine the influence of environmental factors and xenobiotics on seed germination and seedling growth [38,39,40] and to study allelopathic effects in plants [41,42,43,44]. The thermal power emitted by plant tissue and the total amount of emitted energy is directly proportional to tissues’ metabolic activity [45]; they reflect tissue viability and its response to stressors [46]. The curves of specific thermal power obtained for the S or PN-treated maize seedlings were linear, similar to the control seedlings, but of much lower intensity of metabolic changes, expressed by lower values of emitted heat. On the other hand, the curves of specific thermal power for the barnyard grass treated with PNs were of lower values of emitted heat and differed in shape compared to seedlings of control and treated with surfactants. The lowest values were recorded for the 5% concentration of the peppermint oil (PN4) treatment, indicating that the higher the peppermint oil concentration in the emulsion, the more inhibitory effects on seed germination and seedling growth. To the best of our knowledge, our results are the first to show the changes in heat emission in the maize and barnyard grass seedlings treated with the PNs but are consistent with those of [43,44,47], who found that bioactive compounds from allelopathic plants quantitatively and qualitatively change the heat emission patterns of the acceptor seedlings. 

A detailed insight into the chemical composition (qualitative) changes caused in the endosperm of maize and barnyard grass seedlings under S, and PNs treatments were tested by the FT-Raman spectroscopy, which allows the characterization of the chemical composition of the tested material. It is a non-destructive method to study the effects of biotic and abiotic stresses on plants [48,49], so it can be successfully used for studies on living plant tissues [50,51,52]. After seed imbibition, catabolic processes start germination. The storage materials in seeds are decomposed in the early stages of germination, and this process is usually fast. Both in maize and barnyard grass, the storage materials are carbohydrates. Therefore, the profile of these compounds is primarily visible in the spectra of the tested objects. The height of the bands in the spectrum is proportional to the content of compounds in the tissue [53]. The obtained spectra showed that the peppermint oil in each of the PNs concentrations accelerated the breakdown of polysaccharides into monosaccharides in the initial seed germination stage as their increase was observed. This may point to the decomposition of storage materials, which is concurrent with the effects observed in other works, e.g., of essential hemp oil on wild oat and cornflower seedlings [54], and herbal extracts from arnica, ribwort, hypericum, milfoil, sunflower, and sage on seedlings of mustard and oilseed rape [47]. Additionally, a hierarchical analysis of similarity proved significant differences in the chemical composition of seedlings treated with nanoemulsions with peppermint oil (first group) versus control and surfactants treated plants (second group).

The second experiment tested whether leaf-sprayed nanoemulsions with peppermint display herbicidal effects on maize and barnyard grass. The analysis of effective doses (ED) causing different levels of plant damage turned up promising results as the value of the effective dose causing 10% of maize damage (leaf necrosis) was higher than both effective doses, causing 50% and 90% of the damage of barnyard grass. Moreover, the length and dry weight of aerial parts and relative water content (RWC) of maize were similar to control and PN treatments up to 1.5%. In contrast, there was a significant drop of these traits already at PN treatment equal to 1% for barnyard grass. The RWC is a good indicator of stress levels in plants because it measures the water status of plant tissues [55]. Usually, allelopathic stress causes the reduction of the RWC value (total water potential of a cell), which results in a reduction in plant growth. Correct values of RWC in turgid and transpiring leaves are about 98%, while in severely desiccated and dying leaves they are about 40%. In most crop species, the typical RWC at wilting is around 60% to 70% [56]. In our experiment, the RWC values of maize and barnyard grass treated with the increasing doses of PNs were dropping gradually; in barnyard grass treated with PN5, the RWC was about 60%, which is the value on the verge of dying out of plant tissue [56]. 

Additionally, 72 h after treatments, the measurement of Chlorophyll *a* (Chl *a*) fluorescence kinetics, a non-invasive and non-destructive method [57,58], was used to analyze the photosynthetic apparatus’ response to the leaf-treatments of maize and barnyard grass. This method allows us to measure, in a short time, the efficiency of absorption, distribution, and dissipation of light energy in photosystem II (PSII) in reaction to external factors [48,49] that inform of, e.g., the productivity of crops. Interestingly, two fluorescence parameters responsive to stress in plants, namely the maximum quantum yield of the photosystem II (Fv/Fm) and the maximum quantum yield of primary photochemistry (Fv/F0) [58], decreased significantly for both maize and barnyard grass only in plants sprayed with the two highest doses of PNs. A similar situation was for the parameters that describe the specific energy fluxes calculated per reactive center (RC), which statistically increased in maize only when treated with PN5 and in barnyard grass for PN4 and PN5 treated plants. The major difference in the Chl *a* fluorescence parameters between maize and barnyard grass was noted for the phenomenological fluxes per cross-section (CS) parameters and the performance index (PI), which decreased for all the S and PNs treatments in barnyard grass. Compared to the control, the parameter values converted to the excited photosynthetic surface area of the sample (CSm) decreased, while the values converted to the reactive reaction center (RC) increased. This may be related to the number of reactive reaction centers because not all reaction centers on the excited surface area were active [57]. The PI reflects the functionality of photosystems I and II and provides quantitative information on the current state of plant performance in response to stressful conditions, which is why it is a good indicator to investigate the plant’s overall photosynthetic efficiency under different abiotic conditions stresses [59]. These results point to a higher susceptibility of barnyard grass to the leaf-sprayed PNs than maize. 

## 4. Materials and Methods

### 4.1. Chemical Composition of Peppermint Essential oil Used in the Study

The peppermint (*Mentha × piperita* L.) essential oil of southern Europe was purchased (Avicenna Oil, PL). The EO was analyzed by gas chromatography coupled with mass spectrometry (GC-FID-MS). Apparatus: Trace GC Ultra gas chromatograph coupled with DSQ II mass spectrometer (Thermo Electron Corporation), non-polar capillary column Rtx-1 ms (60 m × 0.25 mm, 0.25 m film thickness), programmed temperature: 50 (3 min)—300 °C, 4 °C/min, injector (SSL) temperature 280 °C, detector (FID) temperature 300 °C, transfer line temperature 250 °C, carrier gas–helium, flow with constant pressure 200 kPa, split ratio 1:20. The mass spectrometer parameters were ion source temperature 200 °C, ionization energy 70 eV (EI), scan mode: full scan, mass range 33–420. The percentages of constituents were computed from the GC peak area without using a correction factor. The components were identified based on comparing their mass spectra with those in [60] and computer libraries: NIST 2011, and MassFinder 4.1. Additionally, laboratory (RI_lab_) and literature retention indices (RI_lit_) collected in the Institute of Natural Raw Materials and Cosmetics database of the Lodz University of Technology were compared to confirm identification. The laboratory linear retention indexes were determined regarding a series of n-alkanes C8–C24.

### 4.2. Nanoemulsion (PN) Preparation and Physicochemical Characteristics

The nanoemulsions (PNs) used in this study consisted of peppermint oil, Eco Tween 80 (Croda, Poland), a 100% bio-based ethoxylated sorbitan ester-based on a natural fatty acid (oleic acid), as a surfactant, and deionized water. In two stages, all formulations were prepared by the high-energy emulsification method (ultrasonification). The first step was the preparation of the pre-emulsion by combining the aqueous phase with the mixture of the essential oil and surfactant at T ≤ 40 °C, under magnetic stirring (v = 300 rpm). Then, the coarse emulsion was processed with a probe-type sonicator (UP200 Ht, Hielscher) with a maximum power output of 200 W. 

In the studies, the central-composition design was applied. It was generated in the Statistica^®^ ver. 13 software (TIBCO Software Inc., Palo Alto, CA, USA). The group of input parameters included: essential oil and concentration, sonification time, and amplitude. The ranges of the variability of the process independent variables were as follows:

- essential oil concentration (%): 1, 3, 5;
- emulsifier concentration (%): 1, 3, 5;
- amplitude (%): 69, 79, 89
- sonification time (min.): 1, 2, 3.

The output parameters (dependent variables) included average droplet size, polydispersity index, viscosity, and stability after 2 weeks. Supplementary Table S3 presents the specific values of each process parameter and the analytical results.

The PNs’ mean droplet diameter (Z-Ave d.nm) and polydispersity index (PDI) were measured with a Dynamic Light Scattering (DLS) method (Zetasizer Nano ZS, Malvern Instruments, Malvern, UK) at 25 °C. The samples were diluted with deionized water 1:10 (*v*/*v*) to diminish the opalescence before the measurement and avoid multiple scattering effects. The analysis was performed three times (*n* = 3) for each sample to determine the droplet size mean values and standard deviation.

The rheological properties of the obtained formulations were conducted using a rotational rheometer (Brookfield R/S-CPS Plus Rheometer, Harlow, UK), at room temperature (25 °C), in a shear rate range up to 500 s^−1^.

Stability tests of the obtained samples were examined with the centrifuge method, the test of variable temperatures, and the examination of the size change over time. The sample was subjected to centrifugation at 3500 rpm for 15 min and then analyzed visually for any phase separation. Next, the prepared nanoemulsions were assessed for storage stability at 5, 25, and 40 °C for 30 days. The visual transitions from steady-state to creaming and coalescence for 30 days were noted. The changes in droplet size were measured by the DLS method. 

The morphology and size of the PN particles were investigated using a transmission electron microscope JEOL JEM 2100 HT (Jeol Ltd., Tokyo, Japan). Selected samples were collected on 300 mesh grids made from copper covered with formvar film; 5 µL of samples were applied on each grid. The excess was removed using filter paper and was left to dry at ambient temperature. The electron microscope was used for observation at an accelerating voltage of 80 kV. Images were taken using a 4 k × 4 k camera (TVIPS) equipped with EMMENU software ver. 4.0.9.87 (TVIPS GmbH, Gauting, Germany).

### 4.3. Metabolic Activity of Maize and Barnyardgrass Seedlings Treated with PNs

Metabolic activity during the growth of maize (cv. Lokata) and barnyardgrass (*Echinochloa crus-galli*) seedlings was measured at 20 °C using an isothermal calorimeter TAM III equipped with TAM Assistant Software (Thermo Activity Monitor, TA Instruments, New Castle, DE, USA) according to [47]. Briefly, three two-day-old seedlings of maize pre-germinated on the distilled water were placed into a 25 mL calorimetric ampoule with 400 μL of PN (concentration: 1%; 1,5%; 2% or 5%) or distilled water (control) at the bottom. The reference ampoule contained 400 μL of PN in an appropriate concentration or water (control) only. For barnyard grass, five seven-days-old seedlings pre-germinated on the distilled water were placed into 4 mL calorimetric measuring ampoule with 40 μL of PN (concentration: 1%; 1,5%; 2% or 5%) or water (control). Thermal power curves were recorded for 24 h. The measurements for each PN concentration and the control were taken in fifteen biological repetitions for barnyard grass and six for maize (one biological repetition was the one measurement of seedlings in an ampoule).

### 4.4. FT-Raman Spectroscopy of Maize and Barnyardgrass Seedlings Treated with PNs

The Raman spectra of lyophilized seedlings of maize and barnyard grass were recorded using a Nicolet NXR 9650 FT-Raman Spectrometer (Thermo Scientific, Walthman, MA, USA) equipped with an Nd:YAG laser (1064 nm) and a InGaAs detector. The measurements were performed at room temperature in the range of 400 to 2000 cm^−1^ with a laser power of 0.5 W (64 scans per spectrum), at a spectral resolution of 8 cm^−1^ using an unfocused laser beam approximately 50 μm in diameter and aperture of 80 according to the method described by [39]. The Raman spectra were registered and processed using the Omnic/Thermo Scientific software program (Thermo Scientific, Walthman, MA, USA). Six spectra from different plants were collected and averaged for each plant species and treatment. The spectra were baseline corrected. A hierarchical cluster analysis (similarities between FT-Raman spectra) was performed using Statistica ver. 13.3 (TIBCO Software Inc., Palo Alto, CA, USA) for the whole wavenumber range. The spectral distances were calculated using Ward’s algorithm.

### 4.5. Herbicidal Effects of Leaf-Sprayed PNs on Maize and Barnyard Grass

A pot experiment was carried out in a naturally ventilated vegetation hall with access to sunlight. In each series, pots with a diameter of 11 cm and a capacity of 0.5 L were filled with local sandy soil (pH 5.7). Next, two kernels of maize (cv. Lokata, breeder HR Smolice, PL) or a few seeds of barnyard grass were sown in each pot. After barnyard grass emergence, the seedlings were thinned to five per pot. Pots were watered according to their needs. When plants reached the three-leaf growth phase they were sprayed on 21 June 2021. 

The sprays included:

- Control pots (C), plants sprayed with distilled water only;
- Herbicide control (H), plants sprayed with a commercial mixture of herbicide foramsulfuron + iodosulfuron-methyl sodium + thiencarbazone methyl (39.4 + 1.25 + 12.5 g ha^−1^; MaisTER Power 42.5 OD, Bayer CropSci, PL);
- Two surfactant-only sprays at the dose of surfactant: 1% (S1) and 10% (S5);
- Five doses of PNs: 1% (PN1), 1.5% (PN2); 2% (PN3); 5% (PN4), and 10% (PN5).

Sprays for control C, surfactants S1 and S5, and nanoemulsions NP1-NP5 were applied in the amounts corresponding to 0.022 L m^−2^. The sprayings were carried out using a dark glass atomizer with a graduation of 20 mL.

Seven days after spraying, the visual assessment of damages (tissue necrosis) on the aerial parts of the plants was estimated on a percentage scale (0–100%) that considers the area evaluated. The roots were removed from the soil and rinsed. The length of the aerial parts (from the base of the stalk to the top of the youngest leaf) and the roots of plants were measured. After that, the aerial parts and, separately, roots of each maize plant, and whole barnyard grass plants, were placed in a dryer at105 °C for 24 h and then weighed.

#### 4.5.1. Chlorophyll *a* Fluorescence Measurements

Seventy-two hours after spraying the measurements of maize and barnyard grass leaves, Chlorophyll *a* fluorescence was performed using a Plant Efficiency Analyser (PEA, Hansatech Ltd., Pentney, UK) to describe the efficiency of photosystem II (PSII). The measurements were taken in the central part of mature leaves and adapted to the dark for 30 min using special clips [61]. The following parameters of phenomenological energy fluxes were calculated from the fluorescence curve: the energy absorption by the antenna pigments (ABS/CSm), the amount of energy that was trapped in the reaction center (TRo/CSm), the energy flux for the electron transport (ETo/CSm), and the dissipation of energy as heat (DIo/CSm) where CS is the sample cross-section. The same parameters were also calculated for the reaction centre (RC) and named specific energy fluxes. Moreover, the maximum quantum yield of the photosystem II primary photochemistry (Fv/Fm ratio), the maximum quantum yield of primary photochemistry (Fv/F0), as well as the PSII performance index (PI) was calculated. The detailed equations for the specific parameters are given based on [62]. The measurements were performed in 15 biological replicates for each plant and treatment (1 biological replicate—1 individual leaf).

#### 4.5.2. Relative Water Content (RWC)

Additionally, 72 h after spraying plants in the I series, the leaf fragments of maize and barnyard grass (approximately 20–30 mg) were cut from the central portion of a fully developed leaf and weighed (fresh mass—FM). Next, the leaf fragments were placed in separate vials with 50 mL of water and shaken (WL-972, JW Electronic, Warsaw, Poland) at 20 °C for 24 h. Then, the leaf fragments were weighed again to determine the turgid mass (TM), dried for 24 h at 105 °C, and determined the dry mass (DM). The relative water content (RWC) was calculated using the equation [63]:

RWC [%] = [(FM − DM)/(TM − DM)] × 100%,(1)

where: FM—fresh mass; DM—dry mass; and TM—turgid mass.

The results are the mean value of 10 replicates (from 10 independent plants) for each cultivar and treatment.

#### 4.5.3. Statistical Analyses

The morphometrical and physiological data of plant material were analyzed by the one-way analysis of variance (ANOVA), and means were separated by Duncan’s posthoc test at a significance level of *p* ≤ 0.05 in Statistica ver. 13.3 software (Tibco Software Inc., Palo Alto, CA, USA). The percentage scale (0–100%) of plant damage after PNs treatments was used to estimate the effective doses (ED), causing 10 and 50% damage to maize and 50 and 90% damage to barnyard grass. The ED values were calculated using the concentration–response log-logistic analysis in the *drc* package [64] in the RStudio 2022.02.0 Build 443 software (Prairie Trillium, USA).

## Figures and Tables

**Table 1:** The composition of peppermint essential oil.

Compounds	RI Lit ^1^	RI Lab ^2^	OE ^3^ [%]	EP ^4^
α-Pinene	936	927	0.2	
Camphene	950		t ^5^	
Sabinene	970	963	0.2	
β-Pinene	974	966	0.5	
Octan-3-ol	981	977	t ^5^	
*p*-Cymene	1018	1009	0.6	
1,8-Cineol	1025	1018	5.6	
Limonene	1025	1019	5.6	3.5–14.0
γ-Terpinene	1055	1052	0.3	
Linalool	1086	1086	0.1	
Menthone	1136	1138	27.4	14.0–32.0
Isoborneol	1142		t ^5^	
Isomenthone	1146	1145	4.6	1.5–10.0
Neomenthol	1156	1153	2.5	
Menthol	1172	1166	44.2	30.0–55.0
Isomenthol	1176	1176	0.2	
Pulegone	1215	1215	0.9	0–4.0
Piperitone	1226	1228	0.5	
Neomenthyl acetate	1263	1262	0.4	
Menthyl acetate	1280	1280	6.3	2.8–10.0
Isomenthyl acetate	1298	1294	0.3	
β-Bourbonene	1386	1386	0.2	
β-Elemene	1389	1389	0.1	
(*E*)-β-Caryophyllene	1421	1418	0.1	
Spathulenol	1572	1570	0.3	
Caryophyllene oxide	1578	1574	1.3	
Globulol	1589	1585	0.1	
Total identified			98.2	

^1^ RI lit—literature retention index; ^2^ RI lab—experimental retention index; ^3^ OE—peppermint essential oil; ^4^ EP—European Pharmacopeia 7.0 requirements [25]; ^5^ t—trace amounts t < 0.05.

**Table 2:** Physicochemical properties of selected nanoemulsions containing different peppermint oil concentrations.

Nanoemulsion (*% v/v*) ^1^	Z-Ave (d.nm)/PDI after 24 h	Z-Ave (d.nm)/PDI t = 24 h, after 30 Days	Z-Ave (d.nm)/PDI after Stability Tests
PN-1/(1.0/1.0)	110 ± 2.0/0.055 ± 0.015	111 ± 3.0/0.068 ± 0.006	109 ± 3.0/0.073 ± 0.005
PN-2/(1.5/1.5)	104 ± 1.0/0.048 ± 0.012	107 ± 3.0/0.059 ± 0.009	106 ± 2.0/0.039 ± 0.016
PN-3/(2.0/2.0)	99 ± 1.0/0.043 ± 0.012	101 ± 2.8/0.040 ± 0.010	103 ± 2.0/0.038 ± 0.002
PN-4/(5.0/5.0)	76 ± 0.8/0.099 ± 0.009	81 ± 0.6/0.046 ± 0.008	77 ± 2.0/0.073 ± 0.022

^1^*v*/*v*—volume of surfactant to the volume of peppermint oil; Z-Ave (d. nm)—mean droplet diameter (nm); PDI—polydispersity index.

**Table 3:** Vibrational bands and their assignments for maize and barnyard grass.

Band	Vibrational Mode	Assignment
357	skeletal modes of the glucose pyranose ring	carbohydrates [26]
440	skeletal modes of the pyranose ring	carbohydrates [27]
478	CCO and CCC deformations; related to glycosidic ring skeletal deformations δ(C−C−C) + τ(C−O). Scissoring of C−C−C and out-of-plane bending of C−O	carbohydrates [27]
577	ν(C−O−C) Glycosidic	carbohydrates [27]
713	δ(C−C−O) related to glycosidic ring skeletal deformations	carbohydrates [27]
768	δ(C−C−O)	carbohydrates [27]
865	δ(C−C−H) + δ(C−O−C) glycosidic bond; anomeric region	carbohydrates [27]
939	δ(C−O−C) + δ(C−O−H) + ν(C−O) α-1,4 glycosidic linkages	carbohydrates [27]
1051	ν(C−O) + ν(C−C) + δ(C−O−H)	cellulose, lignin [28]
1083 (1084)	ν(C−O) + ν(C−C) + δ(C−O−H)	carbohydrates [27]
1126 (1125)	ν(C−O) + ν(C−C) + δ(C−O−H)	carbohydrates [27]
1262 (1265)	δ(C−C−H) + δ(O−C−H) + δ(C−O−H)	carbohydrates [27]
1340	ν(C−O); δ(C−O−H)	carbohydrates [27]
1380 (1379)	δ(C−O−H)—coupling of the CCH and COH deformation modes	carbohydrates [27]
1460 (1459)	δ(CH_2_) + δ(CH_3_); δ(CH) + δ(CH_2_) + δ(C−O−H) CH, CH_2_ and COH deformations	aliphatic [29] carbohydrates [27]
1602	ν(C−C)ring + σ(CH)	lignin [30]
1655	ν(C=O)	amide I α-helix [31]

**Table 4:** Effective doses (ED) of peppermint nanoemulsions (% content of peppermint oil and surfactant ± standard deviation) causing 10, 50, and 90% of necrosis as assessed for maize and barnyard grass based on the *drc* analysis.

Effective Dose ^1^	Maize	Barnyard Grass
ED10	2.17 ± 1.19	--
ED50	10.3 ± 6.07	1.09 ± 0.05
ED90	--	1.67 ± 0.19

^1^ Effective dose (ED): an estimated dose causing 10%, 50%, or 90% damage to the aerial parts of plants.

## Data Availability

Not applicable.

## Supplementary Materials

The following supporting information can be downloaded at: https://www.mdpi.com/article/10.3390/molecules27113480/s1, Table S1. Chl *a* fluorescence parameters for control maize leaves and treated with herbicide, surfactants and PNs. Mean values marked with the same letters did not differ significantly at *p* ≤ 0.05 according to Duncan’s test, *n* = 15. The percentage values of changes compared to the control plants (taken as 100%) are given in parenthesis. Table S2. Chl *a* fluorescence parameters for control barnyard grass leaves and treated with herbicide, surfactants, and PNs. Mean values marked with the same letters did not differ significantly at *p* ≤ 0.05 according to Duncan’s test, *n* = 15. The percentage values of changes compared to the control plants (taken as 100%) are given in parenthesis. Table S3. Matrix of the experimental design and experimental data obtained for the dependent variables. Figure S1. The representative photos of damages caused to maize (A) and barnyard grass (B) seven days after leaf-spraying with the peppermint nanoemulsions. C – water control; H – herbicide; PN1-PN5 – peppermint nanoemulsions.

## Sample Availability

Samples of the compounds are not available from the authors.
