# Supplementary material for: Bio-Herbicidal Potential of Nanoemulsions with Peppermint Oil on Barnyard Grass and Maize
Source: Molecules. 2022 May 28;27(11):3480. doi: 10.3390/molecules27113480 (PMC9181968; doi:10.3390/molecules27113480)
Supplement: Supplementary file 1 [file molecules-27-03480-s001.zip › molecules-1723903-supplementary.pdf]

Tabele S1. Chl *a* fluorescence parameters for control maize leaves and treated with herbicide, surfactants and PNs. Mean values marked with the same letters did not differ significantly at $p \leq 0.05$ according to Duncan's test, n = 15. The percentage values of changes compared to the control plants (taken as 100%) are given in parenthesis.

**parametrs**	**maize**								
	**C**	**H**	**S1**	**S5**	**PN1**	**PN2**	**PN3**	**PN4**	**PN5**
**Fv/Fm**	0.798^{ab}	0.785^{abc} (-1.6)	0.798^{ab} (0)	0.805^{a} (+0.9)	0.796^{abc} (-0.3)	0.798^{ab} (0)	0.789^{abc} (-1.2)	0.777^{c} (-2.6)	0.784^{bc} (-1.7)
**Fv/Fo**	4.000^{a}	3.729^{ab} (-6.8)	3.999^{a} (0)	4.150^{a} (+3.7)	3.941^{ab} (-1.5)	4.013^{a} (+0.3)	3.771^{ab} (-5.7)	3.515^{b} (-12.1)	3.684^{ab} (-7.9)
**ABS/RC**	1.939^{bc}	2.028^{b} (+4.6)	1.916^{bc} (-1.2)	1.962^{bc} (+1.161)	1.899^{c} (-2.1)	1.856^{c} (-4.3)	1.933^{bc} (-0.3)	2.029^{b} (+4.6)	2.147^{a} (+10.7)
**DIo/RC**	0.393^{cd}	0.435^{abc} (+10.9)	0.387^{cd} (-1.4)	0.383^{d} (-2.6)	0.389^{cd} (-1.0)	0.375^{d} (-4.5)	0.409^{bcd} (+4.2)	0.453^{ab} (+15.4)	0.464^{a} (+18.0)
**TRo/RC**	1.547^{bc}	1.592^{b} (+3.0)	1.528^{bc} (-1.2)	1.579^{b} (+2.1)	1.510^{bc} (-2.4)	1.481^{c} (-4.2)	1.524^{bc} (-1.5)	1.576^{b} (+1.9)	1.683^{a} (+8.8)
**ETo/RC**	0.764^{ab}	0.736^{b} (-3.7)	0.745^{ab} (-2.5)	0.770^{ab} (+0.8)	0.754^{ab} (-1.4)	0.757^{ab} (-0.9)	0.774^{ab} (+1.2)	0.741^{ab} (-3.0)	0.798^{a} (+4.4)
**ABS/CSm**	1112^{ab}	1122^{ab} (+0.9)	1160^{ab} (+4.3)	1131^{ab} (+1.6)	1160^{ab} (+4.2)	1187^{ab} (+6.7)	1202^{a} (+8.0)	1140^{ab} (+2.5)	1100^{b} (-1.1)
**DIo/CSm**	225^{ab}	242^{ab} (+7.6)	234^{ab} (+4.2)	221^{b} (-1.6)	236^{ab} (+5.1)	237^{ab} (+6.3)	253^{a} (+12.8)	253^{a} (+12.7)	238^{ab} (+5.9)
**TRo/CSm**	888^{ab}	881^{ab} (-0.8)	926^{ab} (+4.3)	909^{ab} (+2.4)	924^{ab} (+4.0)	948^{a} (+6.8)	948^{a} (+6.8)	887^{ab} (-0.1)	862^{b} (-2.9)
**ETo/CSm**	441^{abc}	406^{c} (-7.8)	453^{abc} (+2.9)	444^{abc} (+0.7)	465^{ab} (+5.4)	487^{a} (+10.5)	483^{a} (+9.6)	421^{bc} (-4.4)	409^{bc} (-7.2)
**PI**	2.053^{a}	1.586^{b} (-22.8)	2.033^{a} (-1.0)	2.042^{a} (-0.5)	2.153^{a} (+4.8)	2.319^{a} (+13.0)	2.052^{a} (-0.1)	1.604^{b} (-21.9)	1.567^{b} (-23.7)

Tabele S2. Chl a fluorescence parameters for control barnyard grass leaves and treated with herbicide, surfactants and PNs. Mean values marked with the same letters did not differ significantly at $p \leq 0.05$ according to Duncan's test, n = 15. The percentage values of changes compared to the control plants (taken as 100%) are given in parenthesis.

**parametrs**	**Barnyard grass**								
	**C**	**H**	**S1**	**S5**	**PN1**	**PN2**	**PN3**	**PN4**	**PN5**
**Fv/Fm**	0.814[a]	0.808[a] (-0.7)	0.811[a] (-0.3)	0.808[a] (-0.7)	0.804[a] (-1.1)	0.808[a] (-0.7)	0.806[a] (-0.9)	0.814[a] (0)	0.763[b] (-6.2)
**Fv/Fo**	4.379[a]	4.231[a] (-3.4)	4.315[a] (-1.5)	4.231[a] (-3.4)	4.153[a] (-5.2)	4.265[a] (-2.6)	4.180[a] (-4.5)	4.467[a] (+2.0)	3.385[b] (-22.7)
**ABS/RC**	1.819[cd]	1.991[ab] (+9.4)	1.894[bcd] (+4.1)	1.913[bcd] (+5.2)	1.967[abc] (+8.2)	1.954[abcd] (+7.4)	1.956[abcd] (+7.5)	2.072[a] (+13.9)	1.807[d] (-0.7)
**DIo/RC**	0.340[b]	0.382[ab] (+12.6)	0.357[b] (+5.2)	0.368[b] (+8.2)	0.386[ab] (+13.5)	0.377[ab] (+10.9)	0.380[ab] (+11.8)	0.387[ab] (+13.9)	0.428[a] (+26.0)
**TRo/RC**	1.479[cd]	1.608[ab] (+8.7)	1.537[bc] (+3.9)	1.545[bc] (+4.5)	1.582[abc] (+6.9)	1.578[abc] (+6.6)	1.576[abc] (+6.5)	1.686[a] (+13.9)	1.379[d] (-6.8)
**ETo/RC**	0.797[a]	0.776[abc] (-2.7)	0.765[abcd] (-4.0)	0.729[d] (-8.5)	0.767[abcd] (-3.8)	0.750[bcd] (-5.9)	0.790[ab] (-0.8)	0.740[cd] (-7.0)	0.631[e] (-20.8)
**ABS/CSm**	1341[a]	1183[ab] (-11.8)	1209[ab] (-9.9)	977[cd] (-27.2)	1136[bc] (-15.3)	1127[bc] (-16.0)	1188[ab] (-11.4)	839[de] (-37.4)	697[e] (-48.1)
**DIo/CSm**	251[a]	227[a] (-9.4)	228[a] (-8.9)	187[bc] (-25.3)	224[a] (-10.6)	218[ab] (-13.1)	232[a] (-7.5)	155[cd] (-38.3)	149[d] (-40.6)
**TRo/CSm**	1091[a]	956[ab] (-12.3)	981[ab] (-10.1)	790[cd] (-27.6)	912[bc] (-16.4)	909[bc] (-16.6)	956[ab] (-12.4)	685[d] (-37.2)	548[e] (-49.8)
**ETo/CSm**	589[a]	467[b] (-20.7)	491[b] (-16.7)	377[cd] (-36.0)	442[bc] (-24.9)	434[bc] (-26.3)	479[b] (-18.7)	306[de] (-48.1)	258[e] (-56.2)
**PI**	2.857[a]	2.104[bc] (-26.4)	2.331[b] (-18.4)	2.042[bc] (-28.5)	1.994[bc] (-30.2)	2.040[bc] (-28.6)	2.175[bc] (-23.9)	1.765[c] (-38.2)	2.157[bc] (-24.5)

Table S3. Matrix of the experimental design and experimental data obtained for the dependent variables.

Sample	Input parameters				Output parameters			
	C_{Oil} [%]	$C_{Emulsifier}$ [%]	Amplitude [%]	Time	Z- Ave [d.nm]	PDI	Viscosity [mPa·s]	Stability
N17	3%	5%	79	3 min	210±10	0.277±0.50	300	+
N13	3%	3%	69	2 min	301±17	0.476±0.040	250	-
N18	3%	5%	89	2 min	360±20	0.375±0.120	500	-
N23	5%	3%	79	3 min	162±3	0.081±0.035	466	+
N6	1%	3%	89	1 min	45±0.2	0.062±0.006	278.5	+
N16	3%	5%	69	1 min	117±3	0.173±0.011	376	+
N20	5%	1%	79	1 min	-	-	-	-
N21	5%	1%	89	3 min	-	-	-	-
N4	1%	3%	69	3 min	100±12	0.273±0.023	300	+
N14	3%	3%	79	1 min	70 ±2	0.186±0.011	395	+
N26	5%	5%	79	2 min	480±43	0.660±0.170	600	-
N22	5%	3%	69	1 min	134 ±4	0.164±0.0008	480	+
N8	1%	5%	79	1 min	176±32	0.399±0.232	213	-
N9	1%	5%	89	3 min	250.1±50	1.000	238	-
N25	5%	5%	69	3 min	560±60	0.89±0.13	780	-
N10	3%	1%	69	3 min	155±5	0.117±0.02	497.5	+
N11	3%	1%	79	2 min	163 ±4	0.127±0.009	569.5	+
N5	1%	3%	79	2 min	48±0.2	0.164±0.032	365.5	+
N12	3%	1%	89	1 min	161±5	0.166±0.016	313	+
N7	1%	5%	69	2 min	43.41±4	0.134±0.019	422	+
N2	1%	1%	79	3 min	157 ±3	0.150±0.006	239.7	+
N15	3%	3%	89	3 min	203 ±5	0.046±0.005	353.5	+

N24	5%	3%	89	2 min	136 ±2	0.099±0.019	265	+
N3	1%	1%	89	2 min	110.0±3	0.055±0.017	505	+
N27	5%	5%	89	1 min	78± 2	0.111±0.013	330	+
N1	1%	1%	69	1 min	62±3	0.577±0.015	436	-
N19	5%	1%	69	2 min	181±8	0.183±0.043	375	+
N28	3%	3%	79	2 min	85±2	0.107±0.009	305	+

Legend: (+) –sample passed the stability tests, and its size did not change with time; (-) - sample did not pass the stability tests and destabilized.

A.

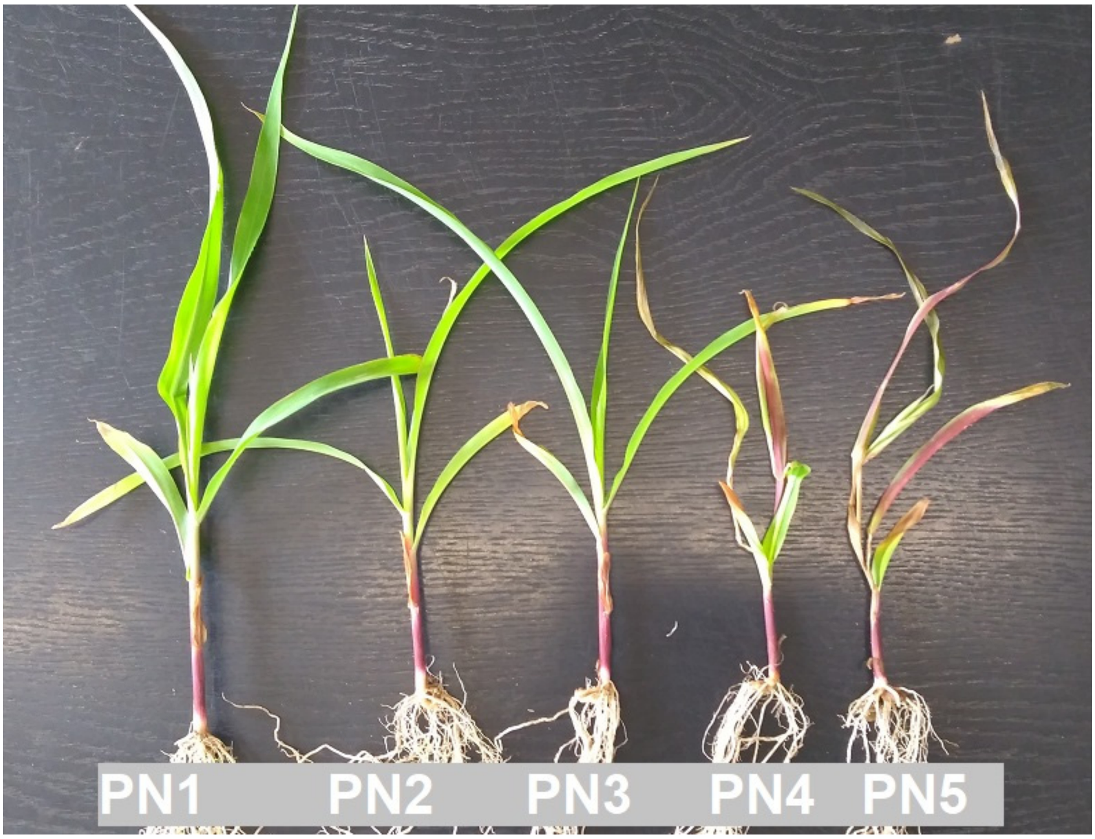

B

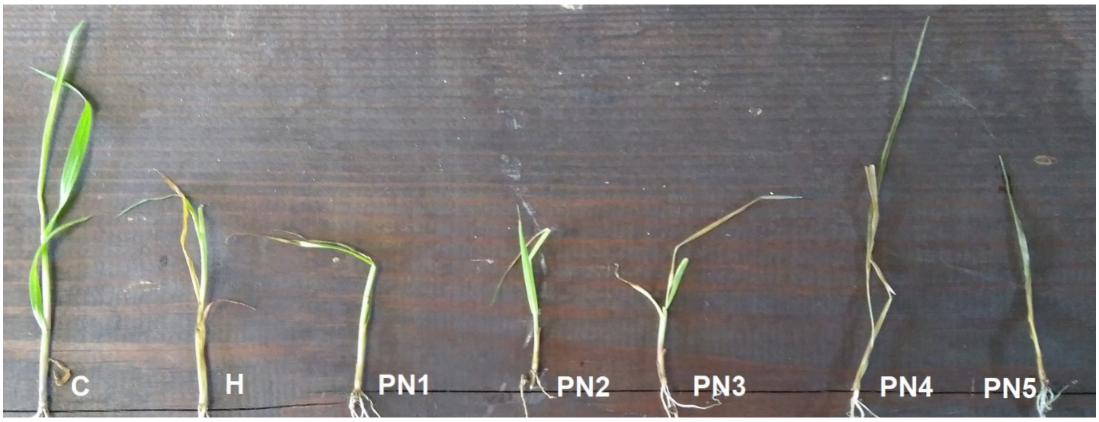

Figure S1. The representative photos of damages caused to maize (A) and barnyard grass (B) seven days after leaf-spraying with the peppermint nanoemulsions. C – water control; H – herbicide; PN1-PN5 – peppermint nanoemulsions.
